# Ophthalmic Biomarkers for Alzheimer’s Disease: A Review

**DOI:** 10.3389/fnagi.2021.720167

**Published:** 2021-09-10

**Authors:** Ayesha Majeed, Ben Marwick, Haoqing Yu, Hassan Fadavi, Mitra Tavakoli

**Affiliations:** ^1^Medical School, University of Exeter, Exeter, United Kingdom; ^2^Imperial College London, London, United Kingdom; ^3^Exeter Centre of Excellence for Diabetes Research, University of Exeter, Exeter, United Kingdom; ^4^National Institute for Health Research, Exeter Clinical Research Facility, Exeter, United Kingdom

**Keywords:** neurodegenerative disease, Alzheimer’s disease, ophthalmic biomarker, oculomics, ophthalmic imaging, dementia

## Abstract

Alzheimer’s disease (AD) is a progressive neurodegenerative disease characterized by neuronal loss, extracellular amyloid-β (Aβ) plaques, and intracellular neurofibrillary tau tangles. A diagnosis is currently made from the presenting symptoms, and the only definitive diagnosis can be done post-mortem. Over recent years, significant advances have been made in using ocular biomarkers to diagnose various neurodegenerative diseases, including AD. As the eye is an extension of the central nervous system (CNS), reviewing changes in the eye’s biology could lead to developing a series of non-invasive, differential diagnostic tests for AD that could be further applied to other diseases. Significant changes have been identified in the retinal nerve fiber layer (RNFL), cornea, ocular vasculature, and retina. In the present paper, we review current research and assess some ocular biomarkers’ accuracy and reliability that could potentially be used for diagnostic purposes. Additionally, we review the various imaging techniques used in the measurement of these biomarkers.

## Introduction

Alzheimer’s disease (AD) is a neurodegenerative disorder that was first described more than a century ago by German psychiatrist Alois Alzheimer (Auguste, 1907; Jarvik and Greenson, 1987). AD usually starts slowly and progressively worsens. The most common early symptom is difficulty in remembering recent events. As the disease advances, symptoms can include difficulties with language abilities, disorientation, mood swings, loss of motivation, self-neglect, and behavioral issues. AD is a degenerative neural disease caused by an abnormal build-up of proteins in the brain that kills cells and damages neurons’ connections. AD, over time, ultimately leads to chronic irreversible and progressive brain cell death (Lim et al., 2016).

While there is no single cause of AD, multiple factors, such as genetics, lifestyle and environment, play important roles (Lim et al., 2016). Variants of the Apolipoprotein E gene (ApoE2, ApoE3, and ApoE4) play an influential role in AD risk, with heterozygosity of the ApoE4 allele increasing a person’s AD risk 16 fold (Martins et al., 2018). Environmentally, traumatic brain injuries and exposure to air pollution have both been shown to increase a person’s risk of developing AD dramatically (Ramos-Cejudo et al., 2018; Peters et al., 2019). Nonetheless, the true cause is likely due to a complex interplay between genetic and environmental factors.

Alzheimer’s disease is the most common form of dementia worldwide, with 850,000 people with dementia living in the United Kingdom (Alzheimer’s Society, 2019’s view on demography). There are over 9.9 million new cases of dementia worldwide each year. Globally, it is estimated around 46 million people have dementia, and this number is expected to increase to over 131 million people by 2050, with AD contributing to between 60 and 70% of cases (Javaid et al., 2016; Lim et al., 2016). In the majority of the affected population, symptoms first appear in their mid-60s which is diagnosed as late-onset type AD (Ames et al., 2013). However, rare, early-onset AD occurs between 30 to mid-60-year olds and can affect people with a family history of the disease (Ames et al., 2013; Markus, 2018).

Depending on the underlying pathology and clinical manifestation of the disease, dementia can be further grouped into AD, Lewy body dementia (DLB), frontotemporal dementia (FTD), and vascular dementia. Neurodegenerative dementias, like AD and dementia with Lewy bodies, are most common in the elderly, while traumatic brain injury and brain tumors are common causes in younger adults.

The true prevalence of AD is unknown. In the United Kingdom, dementia overtook cardiovascular diseases (CVD) as the leading cause of death in 2016 (Office for National Statistics., 2019). Alarmingly, it has been estimated that 50–80% of cases of the most common form of dementia, AD, remain unrecognized in high-income countries due to the challenges in detection and diagnosis (World Alzheimer Report., 2015). Furthermore, it is estimated between 10 and 15% of AD patients are misdiagnosed by specialists, and diagnosis can only be confirmed post-mortem (Thal et al., 2006; Lim et al., 2016).

Successful AD treatments require intervention in the prodromal stages of AD, such as mild cognitive impairment (MCI); thus, biomarkers that enable identification of the prodromal period of AD are crucial (Lim et al., 2016; Kwon et al., 2017). Amyloid-β (Aβ) plaques and neurofibrillary tangles (NFT) pathologies arise decades before symptoms such as cognitive loss, brain atrophy and neurodegeneration are exhibited. This means it is often too late for therapeutic intervention by the time clinical symptoms appear (Hart et al., 2016; Lim et al., 2016; Hane et al., 2017). Currently, there is no modifying therapy for AD (Markus, 2018; Martins et al., 2018; Veys et al., 2019); however, certain medications may help to delay the development of symptoms (Colligris et al., 2018). Currently, there are only two families of medications approved as palliatives for AD symptoms: the cholinesterase inhibitors and the N-methyl-D-aspartate receptor (NMDAR) antagonists, while various new drugs are under clinical trials evaluations, among them the promising new family of secretase inhibitors (Huang et al., 2020).

More recently, the Food and Drug Administration (FDA) approved Aducanumab (Aduhelm^TM^) for the treatment of AD. Aducanumab (an intravenously infused antibody) can clear out clumps of protein known as Aβ plaques linked to AD in the brain (Mullard, 2021). However, there is an ongoing debate and skepticism regarding its efficacy.

However, it has been advised that the clinical trials data did not conclusively show that the drug can reduce cognitive decline in AD patients (Fillit and Green, 2021; Mullard, 2021). Conversely, a study showed that after 1 year of intravenous infusions of Aducanumab in patients with prodromal or mild AD, the drug reduced brain Aβ in a dose and time-dependent manner (Sevigny et al., 2016). This was associated with slowing clinical decline measured using mini-mental state exams (MMSE) (Sevigny et al., 2016). Indeed, there is a need for further research into the efficacy of Aducanumab.

Interest in ophthalmic biomarkers of AD – the leading cause of dementia, has been fueled by challenges in diagnosing the disease and monitoring the disease progression and the response to therapy. The eye has many similarities to the brain; therefore, it can reveal many neural and systemic disorders. In recent years, the emergence of quantifiable high-resolution imaging modalities, which are non-invasive, rapid, and widely available, provided a new area of ocular-neural imaging. Hence, the newly term of oculomics reflects the importance of ocular biomarkers for diagnosing and monitoring a series of neurodegenerative and systemic diseases.

**FIGURE 1:**
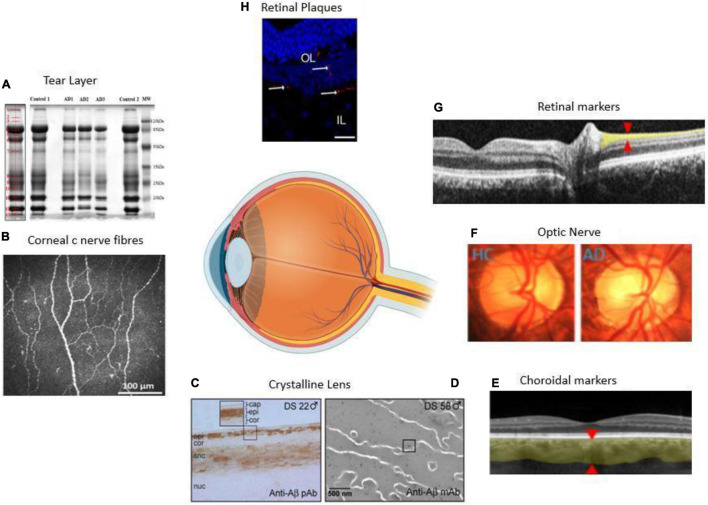
Anatomical locations of ocular biomarkers reviewed in this paper (anti-clockwise from anterior segment to posterior segment). **(A)** Changes in the tear proteome in Alzheimer’s disease (AD). Tear proteins from three AD and two controls are shown by gel imaging. The representative band distribution is demonstrated on the left and was detected with QuantityOne (Kalló et al., 2016). **(B)** Imaging of the cornea using corneal confocal microscopy in a healthy control (HC) demonstrating normal corneal nerve morphology (Tavakoli et al., 2015). **(C)** A lens from a male with Down’s syndrome (DC) showing amyloid-β (Aβ) immunoreactivity in the epithelium, deep cortex, and supranuclear regions. The box shows the magnified detail of the anterior lens (Goldstein et al., 2003). **(D)** Anti-Aβ immunogold electron microscopic analysis of lens from a male with DC. Heterogeneous distribution of anti-Aβ immunoreactive protein aggregates of 5–200 nm localizes heterogeneously within the lens fiber cell cytoplasm (Moncaster et al., 2010). **(E)** Thinning of the choroid demonstrated using optical coherence tomography (OCT) in an AD patient. The red arrowheads denote where the measurements are taken (Lim et al., 2016). **(F)** Optic nerve pallor imaged using a laser Doppler imaging device. The left panel shows the optic nerve of HC, and the right shows the optic nerve of an AD patient (Lim et al., 2016). **(G)** Thinning of the RNFL imaged using OCT. The red arrowheads show where the measurements are normally taken (Lim et al., 2016). **(H)** Immunolabeled retinal slice with anti-Aβ antibodies (red), and Hoechst (blue) to visualize nuclei in late-stage AD in a transgenic mouse (Grimaldi et al., 2018). HC = healthy control, AD = Alzheimer’s disease, Cap = capsule, epi = epithelium, cor = cortex, snc = supranuclear, nuc = nucleus, DS = Down’s syndrome, OL = outer layer, IL = inner layer.

The present review paper aims to review the recent research and assess some ocular biomarkers that could potentially be used for diagnostic purposes. Additionally, we review the various ophthalmic imaging techniques used in the measurement of these biomarkers. Figure 1 summarizes the range of ocular biomarkers at the anterior segment and posterior segment of the eye used for AD diagnosis. We will discuss these biomarkers in detail through this paper.

## Method

For the current paper, the PICO System (P = Problem/Population, I = Intervention, C = Comparison, O = Outcome) was applied to narrow the results and obtain focused literature. Besides, to search for relevant literature, Boolean algorithms were used on four databases – Wiley, Google Scholar, PubMed, and Elsevier. Key terms used included AD OR AD AND diagnosis OR diagnosing AND “early” OR “early-onset” Additional papers were found by going through the reference section for each research paper found. Literature relevant to other neurodegenerative diseases (e.g., Huntington’s disease and multiple sclerosis) were not considered. Overall, for the current paper, 115 primary and secondary published papers were reviewed.

Our literature search was adjusted to include papers between 2006 and 2021. Some exceptions were included outside of these parameters if the research was considered pivotal, once identified from the reference lists of articles identified in the original search.

## Alzheimer’s Disease Pathology and Current AD Diagnostic Techniques

The main pathological hallmarks of AD are intracellular hyperphosphorylated tau (pTau) proteins (pTau) and extracellular deposits of Aβ (Mahajan and Votruba, 2017). The pTau truncates, and self assembles into NFT (Hart et al., 2016). Amyloid precursor proteins (APP) undergo cleavage processes forming either Aβ-40 or Aβ-42 monomers. Aβ plaques are formed when these many monomers of Aβ-40 and Aβ-42 aggregate, forming an insoluble plaque. However, less is known about the process of developing NFT compared to Aβ plaques (Lim et al., 2016).

The amyloid cascade hypothesis of AD pathophysiology states that the deposition of Aβ is the first pathological event; from which NFTs form and neuronal cell death occurs (Ricciarelli and Fedele, 2017). Aβ plaques are known to cause dysfunction of the cross-sectional synaptic network, cognitive decline and progressive brain atrophy (Javaid et al., 2016). The relationship between Aβ plaques and tau are not fully understood. Still, one study discovered that eradication of tau in mice offers protection against some of the toxic effects of Aβ plaques, offering new insight into tau being necessary for Aβ plaques toxicity (Bloom, 2014). Whether these changes observed in the brains of AD patients are reflected in the retinas remains to be fully concluded.

Early diagnosis and disease detection are key in assigning proper treatment therapies (Farah et al., 2018). AD diagnosis is difficult; it is only possible through PET scans of the brain, detecting evidence of amyloid and tau accumulation (Panegyres et al., 2009; Colligris et al., 2018). Furthermore, PET imaging allows for the progression of AD to be tracked antemortem, aiding the disease management (Hane et al., 2017). Even with the advances in technology, it remains difficult for population-wide PET screening due to the high expense, requirement of using radioactive isotypes and inaccessibility of the machines (Hart et al., 2016).

Early detection is vital as numerous delays in the diagnostic system and treatment initiation ultimately decrease treatment success. Normally, diagnostic tests are either blood tests or lumbar punctures. Recently researchers at the University of California found that a new blood-testing technique called Simoa was able to predict AD development by measuring concentrations of pTau-181 in blood plasma. pTau-181 is a modified version of tau previously linked to AD. They showed that pTau-181 in the plasma differed between healthy participants and those with confirmed AD (via autopsies) (National institute of health., 2020). Lumbar punctures can also show early signs of AD as the cerebrospinal fluid (CSF) flows from around the spinal cord and the brain; thus, changes in the levels of amyloid and tau proteins in the CSF can be used as a biomarker to reflect AD-associated pathologic changes in the brain (Alzheimer’s society., 2021). However, it causes discomfort and possible side effects in patients (Farah et al., 2018). Therefore, there is an urge to find accurate, more advanced, and less invasive testing methods.

There is compelling evidence to suggest that specific ocular biomarkers related to neurodegenerative disorders play a pivotal role in the development of retinal impairment or loss of visual function. A range of ocular manifestations of AD, including corneal, retinal, and lens Aβ accumulation, retinal nerve fiber layer (RNFL) loss, and retinal vascular changes, have been proposed as potential biomarkers of the disease. Herein, we examine the evidence regarding the potential value of these ocular biomarkers of AD.

## Discussion

A range of ocular structures present with symptoms in pathological states may be useful as ocular biomarkers or oculomics for detecting and monitoring AD. The eye can be broadly divided into anterior and posterior segments, with this review focussing on the cornea, crystalline lens, and retina. These include pathological changes identified in the retina, such as Aβ plaques and RNFL thickness reduction. There is limited research on the relationship between AD and associated pathological alterations of structures found in the anterior segment of the eye.

Figure 1 and Table 1 summarizes some of these most common biomarkers. In the following sections, we reviewed each of these markers.

**TABLE 1:** Ocular structures and their associated pathological changes in Alzheimer’s disease.

Ocular	Identified associated pathological changes
Structural markers	
Tear fluid	• Changes in the composition of the chemical barrier of tears and proteomics (Gijs et al., 2019; Kalló et al., 2016; Kenny et al., 2019; Wang et al., 2021)
Cornea	• Reduction in sensitivity (Örnek et al., 2015) and increased density of dendritic cells (DCs; Singh and Shilpa, 2020). Reduction of corneal nerve fiber length and density and branch density (Ponirakis et al., 2019; Al-Janahi et al., 2020). The morphological difference in corneal DCs such as greater corneal DC field area and perimeter (Dehghani et al., 2020)
Pupil	• Pupil responses such as greater dilation in single amnestic domain MCI (mild cognitive impairment) compared to controls (Granholm et al., 2017). Increase in pupillary size (Singh and Shilpa, 2020)
Crystalline lens	Abnormal protein deposits present (Tian et al., 2014; Singh and Shilpa, 2020)
Choroid	• Reduction in choroidal thickness (Trebbastoni et al., 2017; Singh and Shilpa, 2020)
Retinal blood vessels	Blood flow disturbance may predate neurodegeneration (Feke et al., 2015). Reduced venous blood flow in the retina (Singh and Shilpa, 2020)
Retina	• Deposition of Aβ (amyloid-beta)plaques and Tau protein (Koronyo-Hamaoui et al., 2011; Koronyo et al., 2017; Singh and Shilpa, 2020). Degeneration of ganglion cells in retina and reduction of the quantity of retinal ganglion cell axons (Singh and Shilpa, 2020)
Optic nerve	• Reduction in optic nerve hemoglobin in AD causing papillary pallor and reduction in thickness of optic nerve (Tsai et al., 1991). Perfusion alterations and axonal loss causing papillary pallor (Bambo et al., 2015). Increase in disc pallor and cup-to-disc ratio (Singh and Shilpa, 2020)
Retinal nerve fiber layer (RNFL)	• Reduction of RNFL thickness (La Morgia et al., 2016; Asanad et al., 2019; Singh and Shilpa, 2020)
Retinal ganglion cell (RGC)	• Degeneration and reduction of RGCs (La Morgia et al., 2016; López-de-Eguileta et al., 2019; Singh and Shilpa, 2020). Reduction of the quantity of retinal ganglion cell axons (Singh and Shilpa, 2020)
**Functional markers**	
Visual acuity	• Reduction in visual acuity (Polo et al., 2017; Salobrar-García et al., 2019; Singh and Shilpa, 2020)
Visual sensitivity	• Dysfunction in different areas of vision and visual cognition (Colligris et al., 2018; Salobrar-García et al., 2019)
Stereopsis/depth perception	• Reduced stereopsis and perception of three-dimensional structures in AD individuals (Lee et al., 2015; Kim and Lee, 2021)
	

## Tear Biomarkers

Tears represent a non-invasive biofluid but are a largely unexplored biomarker source (Glinská et al., 2017). The current understanding of tear film proteomics, including differences in sampling techniques and the knowledge of the core tear proteome, is limited in the literature (Green-Church et al., 2008). The proteomic content of the tear layer has been investigated at some neurodegenerative and inflammatory conditions such as Parkinson disease (Boerger et al., 2019) and multiple sclerosis (Salvisberg et al., 2014; Pieragostino et al., 2019).

There are limited studies that investigated tears as a source of molecular biomarkers for AD.

Tear fluid has proven to be clinically relevant through the discovery of a combination of four tear proteins, namely lipocalin-1, dermicidin, lysozyme C, and lactritin, with a sensitivity of 81% and a specificity of 77% for AD (Kalló et al., 2016). A study by Gijs et al. (2019) suggested the discriminatory power of tear T-tau and Aβ42, as their levels increased in AD patients (Gijs et al., 2019).

MicroRNAs have become an area of particular interest in AD research, with critical roles in the pathogenesis of the disease reported. Total microRNA profusion was found at increased levels in the tear of AD patients, with microRNA-200b-5p as the most promising AD biomarker (Kenny et al., 2019).

Wang et al. (2021) developed a biosensor that could detect Aβ in tear specimens. They concluded the prospect of future screening for AD by using tear biomarkers.

Overall, tears may be a useful novel source of biomarkers for AD as the accessibility and easy sampling of the tear makes it a potential molecular biomarker for AD (Gijs et al., 2019; Lim et al., 2016; Wood, 2016; Tamhane et al., 2019; Karki et al., 2021).

## Cornea

For *in vivo* imaging and examination of AD, the anterior eye is more accessible than the posterior eye. The cornea contains one of the highest concentrations of acetylcholine (ACh) (Dehghani et al., 2018). ACh has a key role in the development and maintenance of corneal epithelia. In AD, there is a deficiency in ACh. Hence, corneal c-nerves fibers and epithelial changes may occur simultaneously to AD neurological changes or severity (Dehghani et al., 2018; Al-Janahi et al., 2020). The cornea is the most densely innervated tissue in the human body (Dehghani et al., 2018). Corneal nerves are derived from the ophthalmic division of the trigeminal nerve and contain both small c-nerve fibers (Figure 1B) and A-delta fibers. The *in vivo* technique of corneal confocal microscopy (IVCCM/CCM) can be used by clinicians and researchers to examine cornea at a cellular level. This method may have the potential to detect early small fiber neuropathy as well as the severity of the disease.

The use of corneal confocal microscopy (CCM) for rapid, non-invasive clinical assessment of corneal nerves has grown substantially, especially in recent years. It has proven to be particularly useful as a diagnostic marker for detecting diabetic neuropathy (Tavakoli et al., 2010a, 2015; Ziegler et al., 2014) and a range of other peripheral neuropathies (Tavakoli et al., 2009, 2010b, 2012; Campagnolo et al., 2013).

**FIGURE 2:**
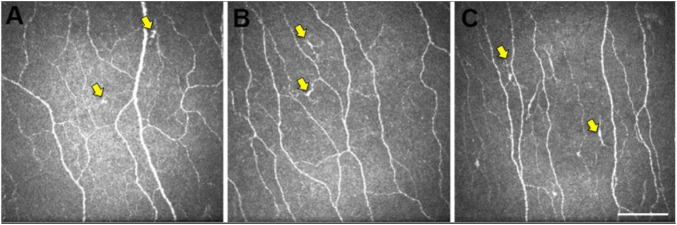
Representative central corneal *in vivo* corneal confocal microscopy (IVCCM) images from the central corneal sub-basal nerve plexus from participants in the control **(A)**, mild cognitive impairment (MCI) **(B)**, and AD **(C)** groups. The epithelial dendritic cells (DCs) (yellow arrows) are smaller and less stratified in CN eyes **(A)** relative to the larger, more stratified morphology of DCs in MCI and AD eyes. Scale bar: 100 μm, applies to all images [image courtesy of reference (Dehghani et al., 2020)].

To our knowledge, there are only three studies that studied the application of CCM to Dementia (Ponirakis et al., 2019; Al-Janahi et al., 2020; Dehghani et al., 2020). However, all those studies studied a small cohort of patients; hence, the results should be interpreted cautiously. Corneal nerve fibers in AD are morphologically different from those in healthy control (HCs) (Figure 2; Ponirakis et al., 2019; Dehghani et al., 2020).

**FIGURE 3:**
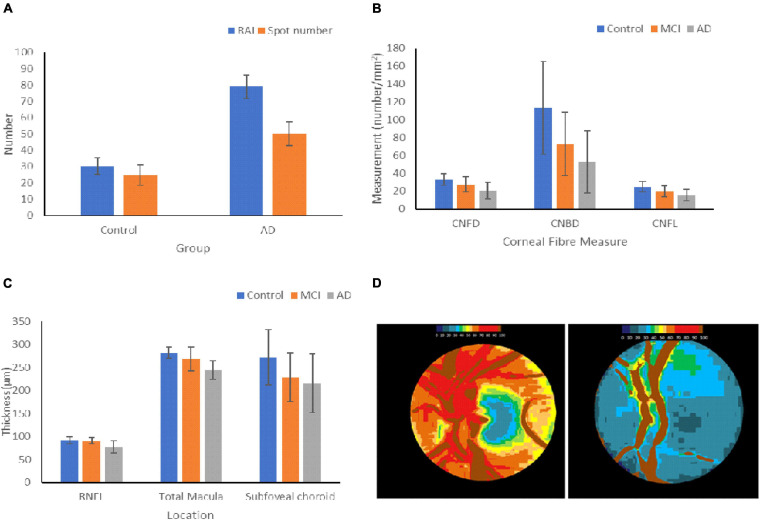
Changes in ocular biomarkers throughout AD progression. **(A)** Aβ Deposition in the retinas of HCs and AD patients. The retinal amyloid index is a quantitative measure of the increase in curcumin fluorescence, and the spot number shows the number of positive curcumin depositions. The graph shows the mean ± standard deviation (Petrella et al., 2019). **(B)** Measurements of the changes in the corneal nerve fibers of HCs and patients with MCI and AD. The graph shows the mean ± standard deviation (Ponirakis et al., 2019). **(C)** Changes in thickness of the RNFL, macula, and subfoveal choroid in HCs, MCI, and AD patients. The graph shows the mean ± standard deviation (Bulut et al., 2016; Kim and Kang, 2019). **(D)** Optic nerve pallor of the left papilla in HC (left) and a patient with AD (right). The images show the changes in hemoglobin absorbance in each papilla, resulting in pallor. Lower hemoglobin absorbances appear blue, and higher absorbances appear in red (Bambo et al., 2015). RAI = retinal amyloid index, CNFD = corneal nerve fiber density, CNBD = corneal nerve branch density, CNFL = corneal nerve fiber length.

Alzheimer’s disease has been shown to affect the corneal nerve fiber length, fiber density, branch density, and dendritic cell (DCs), as shown in Figure 3B (Ponirakis et al., 2019). Ponirakis et al. (2019), in a study of 26 patients with Dementia, showed progressive reduction in corneal nerve fiber length (CNFL) (P < 0.0001) compared to healthy subjects in that study. Al-Janahi et al. (2020), in a study of 66 patients with MCI and dementia, showed the diagnostic accuracy of CCM. They showed the diagnostic accuracy of CCM was high and comparable with the medial temporal lobe atrophy (MTA) rating for dementia but was superior to the MTA rating for MCI.

The research on the potential presence of AD pathological mechanisms in the cornea is limited. Given the different variables that can be measured to assess morphological changes in the corneal nerve fibers, imaging of this ocular structure could be useful in aiding AD diagnosis. However, whilst the limited existing studies seem promising, more thorough research is required in this area to draw more definitive conclusions about the utility of the cornea as a biomarker.

## Pupil

In AD, the pupil has been shown to have reduced latency and amplitude of pupillary light reflex, increased size, and an atypical response to cholinergic antagonists (Singh and Shilpa, 2020). These changes are thought to be associated with the deficiency in ACh found in AD. Pathological degeneration of the Edinger–Westphal nucleus and nucleus basalis of Meynert are considered to be the sources of this deficiency (Singh and Shilpa, 2020). A significant positive correlation has also been found between increased pupillary size and the concentration of Aβ and tau in the cerebral spinal fluid (Frost et al., 2013a). Whilst this could be a potentially useful biomarker, the study had several limitations. Firstly, participants were from a single family with one mutation, so the findings may not apply to other sporadic AD or AD caused by a mutation in a different locus (Frost et al., 2013a). Furthermore, the sample size was small, with only 12 participants sorted into two equal groups), decreasing the reliability of the results (Frost et al., 2013a). Further studies are required to assess the role of pupil changes at AD as a potential marker.

## Crystalline Lens

The human lens contains three parts: lens capsule, the anterior single layer of lens epithelium and lens fibers cells (Figures 1C,D). Like in AD, the aging of the human lens increases misfolded, insoluble protein aggregation and accumulation (Dehghani et al., 2018). This suggest that the lens may not be a very specific AD biomarker.

Frederiske et al. showed that Aβ protein precursor (APP) and amyloid-beta – usually present in the cataractous human lens is toxic to cultured mammalian lens epithelial cells (Frederikse et al., 1996; Dehghani et al., 2018). In another study, Frederikse et al. (1999) also demonstrated that Alzheimer-associated brain pathologic alterations were produced in mice with systemic oxidative stress induced by thiamine deficiency. After analyzing the anti-APPanti-Aβ and fluorescent conjugated secondary antibodies, they found that degeneration of the lens fiber cells was associated with increased local distributions of APP, Aβ and presenilin proteins.

However, some studies have shown a lack of amyloid-beta in the human lens. Ho et al. found very weak non-specific Aβ staining in some cases, but no amyloid deposits or abnormal tau accumulations in the lenses from 11 subjects of AD were detected. Therefore, concluding that AD-related aggregates do not deposit in the eye similar to brain deposits or are present at lower levels or in different forms (Dehghani et al., 2018). This suggests that AD-related aggregates in the lens were not a good marker for aggregates in the brain.

These findings were supported by Williams et al., who found no Aβ deposits or accumulation in any part of the eye, including the lens, after performing hematoxylin and eosin staining and Aβ immunohistochemistry 19 human post-mortem cases (17 with AD and two age-matched controls). Thus, concluding there may be no concurrent or similar AD alterations in the brain and lens (Dehghani et al., 2018).

Whether crystalline lens mirrors brain neuropathological changes and whether amyloid beta accumulates in the lens in preclinical AD is yet to be determined (Dehghani et al., 2018).

## The Retina as a Potential Biomarker

**FIGURE 4:**
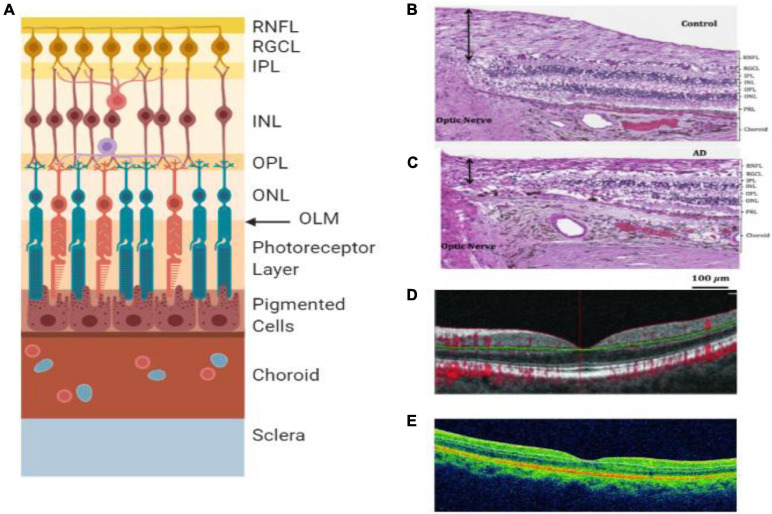
Retinal layers and imaging. **(A)** The individual layers of the retina. **(B)** Light microscopy image of human post-mortem control retina stained with hematoxylin and eosin. The black arrow represents the superior-temporal RNFL thickness (Asanad et al., 2019). **(C)** Light microscopy image of human post-mortem AD retina stained with hematoxylin and eosin. The black arrow represents the superior-temporal RNFL thickness and demonstrates the most thinning close to the optic nerve (Asanad et al., 2019). **(D)** OCT of preclinical AD patient (O’Bryhim et al., 2018). **(E)** OCT of a retina with clinical AD (Cunha et al., 2017). RNFL = retinal nerve fiber layer, RGCL = retinal ganglion cell layer, IPL = inner plexiform layer, INL = inner nuclear layer, OPL = outer plexiform layer, ONL = outer nuclear layer, OLM = outer limiting membrane, AD = Alzheimer’s disease, PRL = photoreceptor layer.

Several studies found that the retina is affected profoundly by AD (Doustar et al., 2017), which may be the reason for visual disorders related to AD (Colligris et al., 2018). The retina harbors the earliest detectable disease-specific signs, which are the Aβ plaques (Figure 1H). It undergoes significant ganglion cell degeneration, thinning the RNFL (Figures 4B,C) and losing axonal projections in the optic nerve (Doustar et al., 2017). The retina is thought to be a part of the central nervous system (CNS) and is the only optically accessible nervous tissue (Doustar et al., 2017). It is much more easily accessible than brain tissue due to the anterior eye’s lucidity compared to the skull (Veys et al., 2019). Figure 4 demonstrates the ten different retina layers. Recently, scientists have developed promising eye-scan methods to discern AD at its earliest stage before the major symptoms occur, leading to improved AD management. Examples include *in vivo* confocal scanning laser ophthalmoscopy (cSLO) imaging where retinal Aβ index correlates with cerebral Aβ plaques; and non-invasive retinal imaging: OCT (Figures 4D,E; Doustar et al., 2017). *In vivo* imaging of retinas provides an easy, non-invasive way to capture the pathologic changes in the brain (Veys et al., 2019).

### Aβ Plaque Deposition/Protein Deposition

Aβ plaques in the retina were first discovered by Koronyo-Hamaoui et al. (2011), where post-mortem eyes of definitive AD and controls of non-AD eyes were processed. This study used imaging of Aβ plaques using curcumin, which binds to the plaques and fluoresces, allowing for *in vivo* retinal imaging (Koronyo-Hamaoui et al., 2011). Koronyo-Hamaoui et al. studied both animal models (mice) and human subjects (Post-mortem eyes and brains). The authors applied a comprehensive methodology including *ex vivo* curcumin staining, intravenous injections of curcumin for *ex vivo* plaque imaging, intravenous injections of curcumin for non-invasive *in vivo* retinal plaque imaging, fluorescence, and bright-field images microscopy. The results indicate that curcumin binds to retinal Aβ plaques in a mouse model of AD and specific curcumin *in vivo*-labeling of Aβ plaques in AD mice and early plaque detection retina. Also, there was a reduction of retinal Aβ plaque burden in AD mice following an immune-based therapy and non-invasive imaging of curcumin-labeled retinal plaques in live AD mice. Furthermore, Aβ plaques in retinal samples from AD patients were identified, and there was the detection of Aβ plaques in retinal samples from suspected early-stage AD patients.

Whole-mounted AD retinas revealed plaques; however, no plaques were detected in the absence of curcumin labeling in the same retinal location. Staining of the varying stages of AD with curcumin and anti-Aβ42 monoclonal antibody showed the abundance of “compacted” Aβ plaques with a dense core globular amyloid deposit and no radiating fibrils (Koronyo-Hamaoui et al., 2011). A smaller proportion of Aβ plaques were the typical central core structure with radiating fibrillar ends of Aβ deposits. This study demonstrates how Aβ plaques can be an ocular biomarker for AD due to the correlation between AD progression/severity and Aβ plaque burden in the retina (Koronyo-Hamaoui et al., 2011). However, this correlation was found in a sample of transgenic mice, so further research is required to determine the validity of this in humans. This study offers another non-invasive method of *in vivo* monitoring of AD progression via optical imaging (Koronyo-Hamaoui et al., 2011). Most of the Aβ plaques were detected from the retina’s inner layers; therefore, it is feasible and easier to replicate this alongside the improvement of ophthalmoscopy technology and equipment. Furthermore, Aβ plaques appear to be an ocular biomarker specific to AD. Therefore, due to this high specificity, Aβ plaques could be used to aid in the differential diagnosis of AD (Tian et al., 2014). This shows promise for using Aβ plaques as an ocular biomarker for AD due to the specificity, as other methods, such as RNFL thickness, can result from dementia and other neurological pathologies, meaning clinically further diagnosis will be required.

One area of consideration is that curcumin does not bind specifically to Aβ plaques; hence it may pose a problem for clinical use (Grimaldi et al., 2018). Curcumin binds to an array of proteins, and a study reported that curcumin binds α-synuclein in Parkinson’s disease and can inhibit pathological aggregation (Jha et al., 2016). Similar research in AD patients found that curcumin can also be used therapeutically parallel to the diagnostic imaging (Mishra and Palanivelu, 2018). Different forms of curcumin were noted to have different affinities, so it may be a solution to test which form of curcumin has the strongest association for Aβ plaques. Curcumin does not bind to non-aggregated Aβ in individuals without AD (den Haan et al., 2018a). However, one criticism is that the study was conducted on brain tissue, which has different morphological Aβ plaques that may affect binding affinity from earlier studies (den Haan et al., 2018b).

The alternative to using curcumin is near-infrared fluorescence ocular imaging (NIRFOI). NIRFOI in mice could capture signals from both insoluble and soluble Aβ. It can differentiate between transgenic AD mice and control mice. Yang et al. (2019) monitored the therapeutic effects of a drug that can alter Aβ levels. This technique could have translational potential for human studies. NIRFOI can capture signals from wide-angle imaging. However, eye color can have a considerable effect on the NIRFOI signal (Yang et al., 2019).

Another study found similar results and measured that the deposits were 5 to 20μm, small and round but were different morphologically to the Aβ plaques observed in AD brain tissue (den Haan et al., 2018b). The plaques were mostly located in the ganglion cell layer (GCL) and inner plexiform layer but with a preference for the superior quadrant, similar to the RNFL studies. La Morgia et al. (2016) identified a lack of or minimal Aβ plaques in HC retinas and an abundance of clusters of Aβ plaques with diverse morphology (compacted and classical) evident in all retinas with AD.

Post-mortem human retinas showed an increase in pTau in the retina’s inner and outer plexiform layers, but no difference in Aβ in AD confirmed patients compared to controls (den Haan et al., 2018b). A wide range of antibodies was used; however, the increased pTau could be caused by other confounding retinal pathology.

Amyloid-β and pTau tangles were found in the retinal ganglion cell layer (RGCL) of pre-symptomatic triple transgenic mice, significantly increasing disease progression (Grimaldi et al., 2018). The presence of protein aggregates in the retina could be used to detect preclinical AD. This technique’s translational potential is not clear and would require submicron lateral resolution, so advances need to be made in high-resolution imaging techniques (Grimaldi et al., 2018). Some studies have used retinal hyperspectral imaging (rHSI) to image Aβ plaques (Hadoux et al., 2019; More et al., 2019). rHSI may have a higher sensitivity in the early stages of AD as the largest spectral deviation from the control group was the MCI patients (More et al., 2019). MMSE scores were used to create the subgroups of AD severity. PET imaging has been used to determine Aβ+ patients and Aβ-controls (Hadoux et al., 2019). There were significant differences in reflectance spectra between individuals with high Aβ, MCI and aged-matched Aβ-controls. This technique was tested and confirmed on a different cohort using a different camera (Hadoux et al., 2019). One theory is the “dynamic biomarker cascade model,” which suggests that each biomarker is effective during a particular disease timeframe (Petrella et al., 2019). This may also happen with retinal biomarkers and need to be considered for future studies.

### Retinal Nerve Fiber Layer

As the retina is thought to be a part of the CNS, it follows that retinal cells may die in neurodegenerative diseases and could lead to retinal thinning. The retina is made of several layers of cells and can be divided into four sectors (Veys et al., 2019). One of the characteristic features of early AD is visual disturbances which may be associated with structural changes such as optic nerve degeneration (Colligris et al., 2018). The RNFL is formed by the expansion of the optic nerve fibers, and it is thickest near the optic disc – gradually diminishing toward the ora serrata (Budenz et al., 2007). RNFL thickness in a healthy eye is 80+ micrometeres (μM) (Budenz et al., 2007).

The changes in RNFL thickness throughout AD progression have been quantified and are shown in Figure 3C.

Although a consensus has not been reached, many studies found that AD patients show significant RNFL thinning (Table 2; Cunha et al., 2017; Asanad et al., 2019; Kwon et al., 2017; Kim and Kang, 2019; López-de-Eguileta et al., 2019). The RNFL naturally decreases with age, which makes it difficult to conclude whether the thinning is due to AD or aging (Kwon et al., 2017).

**TABLE 2:** Summary of main findings of retinal nerve fiber layer changes from published papers.

Publication	Study type	Sample size	Technique	Main finding(s)	Limitations
Parisi, 2003	Cross-sectional	**31** (17 AD, 14 control)	OCT	Reduction in RNFL thickness in AD group compared with healthy control	Small sample size
Berisha et al., 2007	Cross-sectional	**17** (9 mild/moderate probable AD, 8 control)	OCT	RNFL thickness significantly decreased in the AD group in the superior quadrant compared to the control group. No significant differences were reported in inferior, temporal, or nasal RNFL thickness in the groups	Small sample size
Kirbas et al., 2013	Cross-sectional	**80** (40 early untreated AD, 40 control)	SD-OCT	RNFL thickness decreased in the AD group compared to the control group	Relatively small sample size
Marziani et al., 2013	Case-control prospective	**42** (21 AD, 21 control)	SD-OCT	RNFL thickness is thinner in the AD group compared to healthy controls	Spectralis analysis was based on subjective non-automated segmentation
Feke et al., 2015	Cross-sectional	**52** (10 AD, 21 MCI, 21 control)	OCT	No significant difference in RNFL thickness was discovered in AD and control groups	Small sample size
Liu et al., 2015	Cross-sectional	**132** (24 mild AD, 24 moderate AD, 19 severe AD, 26 MCI, 39 control)	OCT	Decrease of RNFL thickness in the superior quadrant in the MCI group compared to controls. This decrease was more significant in the AD group compared to controls	
La Morgia et al., 2016	Cross-sectional	**95** (21 AD, 74 control)	OCT	On average, RNFL thickness in the superior quadrant of the AD group decreased compared to controls. RNFL thickness correlated with age	The small sample size for *in vivo* studies. The study used different inclusion criteria for the two cohorts’ enrolment; thus, selection bias may be introduced
Cunha et al., 2017	Cross-sectional	**202** (50 mild AD, 152 control)	SD-OCT	Decrease of RT thickness in superior pericentral and peripheral sectors in patients with AD. Also, the reduction in global and temporal superior quadrants in pRNFL	Lack of inclusion of amyloid markers (e.g., CSF or amyloid imaging) increased diagnostic accuracy during the participant recruitment stage
den Haan et al., 2017	Cross-sectional	**1967** (887 AD, 216 MCI, 864 control)	OCT	Both AD and MCI groups had decreased pRNFL thickness compared to controls. AD group also reported a decrease in total macular thickness	AD individuals were diagnosed based on clinical criteria and not backed up by biomarker tests
Kwon et al., 2017	Cross-sectional	**90** (30 AD, 30 MCI, 30 control)	OCT	Decrease of RNFL thickness in the superior quadrant in the AD group compared to the healthy group	Small sample size and lack of follow up with the subjects
Lad et al., 2018	Case-control	**48** (15 mild/moderate AD, 15 MCI, 18 control)	OCT	No significant difference in RNFL thickness was discovered in AD, MCI, and control groups	Small sample size. Exclusion of some areas of the retina being analyzed by OCT influences the nerve fiber layer thickness
Sánchez et al., 2018	Cross-sectional	**930** (324 AD, 192 MCI, 414 control)	OCT	No significant difference between RNFL thickness in MCI, AD, and control groups	Cross-sectional study design not allowing conclusions regarding RNFL thinning to be drawn. Furthermore, there is higher inter-individual variability in each group related to a less controlled age range in the population
Santos et al., 2018	Longitudinal	**56** (56 suspected AD)	SD-OCT	Participants with preclinical AD had a significant decrease in macular RNFL volume and decreased the inferior quadrant’s thickness	The study observes suspected AD individuals rather than confirmed AD individuals; thus, the results cannot be projected onto individuals with diagnosed AD
Asanad et al., 2019	Post-mortem	**19** (8 AD, 11 control)	OCT	RNFL thickness was significantly thinner in superior-temporally in the peripapillary region in the AD group compared to the control group	Small sample size analyzed, and results were only derived from severe AD cases
den Haan et al., 2019b	Cross-sectional	**93** (23 AD, 70 control)	OCT	No significant difference in pRNFL thickness between AD and controls	Lack of ophthalmological examinations may introduce subjects with pre-clinical changes of glaucoma and diabetic retinopathy
Kim and Kang, 2019	Cross-sectional	**47** (7 mild to moderate AD, 9 severe AD, 14 aMCI, 17 controls)	OCT	The severe AD group had a significant decrease in pRNFL thickness compared to controls. The mild to moderate AD group had a significant reduction in superior RNFL thickness compared to controls	Small sample size and lack of follow up with the subjects for verification of the relationship between the severity of AD and retinal thickness
López-de-Eguileta et al., 2019	Cross-sectional	**32** (6 AD, 26 MCI)	OCT, 11C-PiB PET/CT	Eyes with positive 11C-PiB PET/CT showed a significant reduction in RNFL thickness	Small sample size
van de Kreeke et al., 2019	Cross-sectional	**142** (57 AD, 85 control)	SD-OCT	No significant difference between pRNFL in AD cases and healthy controls	A small sample size limits statistical power
Zabel et al., 2019a	Cross-sectional	**60** (30 AD, 30 control)	SD-OCT	RNFL thickness in the AD group was significantly thinner compared to healthy controls	Small sample size

*11C-PiB PET/CT – 11C-labeled Pittsburgh compound-B with positron emission tomography, AD – Alzheimer’s disease, aMCI – amnestic mild cognitive impairment, CSF – cerebral spinal fluid, MCI – mild cognitive impairment, OCT – optical coherence tomography, pRNFL – peripapillary retinal nerve fiber layer, RNFL – retinal nerve fiber layer, RT – retinal thickness, SD-OCT – spectral-domain optical coherence tomography.*

In one study, in patients with preclinical AD (cognitively normal but Aβ + as shown by a PET scan), the assessment of RNFL thickness with spectral-domain optical coherence tomography (SD-OCT) failed to show significant differences to healthy subjects imaged (van de Kreeke et al., 2019). Therefore, retinal thinning due to neurodegeneration may only be detectable in the later stages of AD (Zabel et al., 2019a). Additionally, RNFL thickness could not distinguish between different cognitive groups; this may be because RNFL thickness is not strongly involved in AD (Sánchez et al., 2018). However, AD patients were significantly older than the controls; therefore, age could be a confounding factor (Sánchez et al., 2018). Different OCT devices have different segmentation methods, axial resolution, scan area, and imaging protocols that can affect the images produced (den Haan et al., 2019a). OCT image quality can greatly affect the variability of RNFL thickness (Sánchez et al., 2018).

In another study, the RNFL and GCL in patients with AD were significantly thinner than controls measured by SD-OCT (López-de-Eguileta et al., 2019). The mean RNFL thickness was significantly thinner in AD patients compared to MCI using SD-OCT (Kwon et al., 2017). Furthermore, in post-mortem retinas, the RNFL was thinner in AD patients than controls (Asanad et al., 2019). The AD retinas came from patients with severe AD and were much older. As a result, RNFL thickness may be better at detecting later-stage AD (Kim and Kang, 2019). In contrast, several studies have shown that thinning in the RNFL is insufficient to distinguish between AD and controls (Sánchez et al., 2018; den Haan et al., 2019a; van de Kreeke et al., 2019; Zabel et al., 2019a).

Kirbas et al. (2013) discovered that RNFL thinning occurs selectively in the superior quadrant in AD participants (76 ± 6.7 μm) compared to the HC group (105 ± 4.8 μm; *p* = 0.001). No significant difference in RNFL thickness was observed in the remaining three quadrants. Other studies have found a similar decrease in RNFL in patients with AD than the HC group, a correlation between deterioration of RNFL and progression of AD and dementia (La Morgia et al., 2016). Liu et al. compared RNFL thickness of healthy participants with MCI and various severity of AD (mild, moderate, and severe). Liu et al. (2015) discovered a significant difference between all groups in the superior and inferior quadrants but not in nasal or temporal quadrants.

Most previous studies are cross-sectional, making it difficult to fully elucidate the utility of retinal thickness (RT) as a biomarker for AD. A significant decrease in macular RNFL volume was detected in adults with preclinical AD over 27 months, compared to HC s (Santos et al., 2018). This is the first longitudinal study within subjects’ changes in preclinical AD. This study concluded that perhaps thinning of the macular RNFL may be the earliest marker of preclinical AD (Santos et al., 2018). In a recent study (Zabel et al., 2019a), peripapillary RNFL thickness in participants with AD and other retinal pathologies were compared against HCs; where the AD group (95.73 ± 13.52 μm) was significantly thinner than the control group (106.30 ± 8.95 μm). This study discovered significance between AD and the control group in all quadrants except the nasal-superior quadrant. This difference is likely to be due to assessment variation when evaluating participants with MMSE. A meta-analysis concluded that RT is decreased in AD and MCI compared to HCs, further confirming that the retinal changes may reflect neurodegenerative changes (den Haan et al., 2017). However, a few studies have not found a noticeable difference in RNFL between the cohorts of control, healthy subjects, preclinical AD and clinical AD (Berisha et al., 2007; Feke et al., 2015; Lad et al., 2018; Sánchez et al., 2018). Reasons are not fully known and will be difficult to be developed into a biomarker if RNFL thinning is not always present during diseased states in most of the population.

Overall, it seems RNFL thinning appears to be a good biomarker for broader and initial diagnosis of neurological pathologies, including AD, Lewy body dementia, Parkinson’s disease, multiple sclerosis, and even non-neuronal conditions such as strokes (Sánchez et al., 2018). However, there has been no exhaustive research into if it is possible to definitively differentiate between any neurodegenerative disease based solely on the location and degree of retinal thinning. Until such research has been carried out, retinal thinning would have to be used in conjunction with other diagnostic tests to provide an accurate diagnosis, despite the huge potential of the technique (Gulmez Sevim et al., 2019; Veys et al., 2019). However, other ocular diseases can lead to RNFL thinning, such as glaucoma and non-glaucomatous optic neuropathy, which is another challenge in addition to the challenges associated with differentiating between multiple neurodegenerative diseases as RNFL may be reduced in all of them.

Figure 3 shows changes in ocular biomarkers throughout AD progression.

### Optical Coherence Tomography

It is well described that AD pathogenesis starts at least 2–3 decades before the AD symptoms appear (Martins et al., 2018). Yet, there is still no simple, non-invasive, inexpensive technique for diagnosing AD without these symptoms (Panegyres et al., 2009; Martins et al., 2018). The current gold standard technique used for AD diagnosis is brain PET which demonstrates a build-up of accumulated Aβ protein within the AD brain (Panegyres et al., 2009; Martins et al., 2018). However, this is expensive, as well as invasive (Martins et al., 2018). It may be difficult to develop new diagnostic tests due to the significant loss of synapses, neurons, and brain tissue by the time AD symptoms occur (Martins et al., 2018). Hence, effective treatment at pre-clinical stages is needed (Martins et al., 2018), as well as a possible screening process that the general public can go through at mid-age (30 to 60-year-olds) to test for early-onset AD. This screening process may also bring new insight into identifying populations at greater risk of developing AD.

Optical coherence tomography is used to assess the structure of the retina, and several studies using this technique have reported an overall thinning of the retina (Veys et al., 2019). Table 2 summarized some of the studies that applied OCT to investigate RNFL changes at AD.

Kwon et al. (2017) compared the RNFL thickness in the eyes of the elderly cohort of subjects with AD or MCI with age-matched healthy people who have no cognitive disabilities as control. MCI is the transition stage from cognitive decline due to aging to severe cognitive decline due to dementia. OCT was used for observing ophthalmic conditions and measuring RNFL thickness via a cross-section. Ninety participants (30 AD, 30 MCI, and 30 healthy) were recruited, and participants’ cognitive states were evaluated using the Korean equivalent of the MMSE and other tests and scales. The superior quadrants had significantly different RNFL thickness in AD and healthy groups (*p* = 0.03) (Kwon et al., 2017).

Recent studies describe a new sensitive imaging test called polarization-sensitive OCT, which can discern birefringence due to microtubule damage associated with AD (Martins et al., 2018). This technique looks promising in identifying AD at early stages as it is thought that the birefringence comes before RNFL thickness decreasing (Martins et al., 2018). Not only has this technique been used in the laboratory on mice; this has been tested on patients with AD, which showed the efficacy of the technique as a biomarker for early-onset AD (Colligris et al., 2018). A study by Cunha et al. (2017) used spectral-domain OCT to compare the thickness of the peripapillary retinal nerve fiber layer (pRNFL) with the overall RT in AD patients with controls. They found that the pRNFL was significantly reduced for the AD group and in the superior temporal quadrant. The RT was also thinner in superior pericentral and peripheral areas S3 and S6 (Cunha et al., 2017). This highlights the biological significance of spectral-domain OCT as a potential adjuvant in the early diagnosis of AD. The findings could show that peripapillary changes are characteristic of AD (Cunha et al., 2017). However, another study found no significant difference in pRNFL thickness between 23 AD patients and 70 controls. The lack of ophthalmological examinations in this study may have introduced subjects with pre-clinical changes of glaucoma and diabetic retinopathy (den Haan et al., 2019b).

### Confocal Scanning Laser Ophthalmoscopy

Another retina imaging technique is cSLO which shows an “*en face*” view of the retina (Veys et al., 2019). This is useful as optic nerve and vasculature changes can be examined. This technique is currently being used as a non-invasive diagnostic imaging test for patients with Parkinson’s disease (Veys et al., 2019). However, if scientists can adapt this technique for testing for AD signs and the OCT scans, it could be a promising way of accurately diagnosing early-onset AD before any symptoms appear, leading to better management of the disease and possibly personalized treatment. These tests should be refined to improve the histology detail seen so that assessment is accurate when assessing vascular networks (Colligris et al., 2018).

## Retinal Vasculature

The retinal vasculature pathologies are likely to reflect cerebrovascular abnormalities due to the retinal and cerebral tissues being derived from a common embryological origin. One study evidenced the venous blood flow of the retina in the AD patient group decreased by 38.6% compared to the HC group. The same study also found that the retina’s venous blood column diameter in AD participants decreased by 13.3% compared to the control group (Feke et al., 2015). A similar study (Berisha et al., 2007) with nine participants with AD (ranging from mild to moderate probable) found a decrease of 11.2% in the blood column diameter of the major retinal temporal vein compared to the control group. Furthermore, a significant narrowing of the venous blood column can account for the decrease in venous blood flow rate (Berisha et al., 2007).

Aside from the retinal vasculature narrowing and causing reduced blood flow, Michael Williams et al. (2015) evidenced that from a 507 participants case-control study, participants with AD were more likely to have altered microvasculature of the retina. This leads to a sparser retinal microvascular network, and this could be mirroring similar alterations occurring in the cerebral microcirculation (Williams et al., 2015). Similar results were also obtained by another study (Frost et al., 2013b), and altogether, these studies suggest that retinal vasculature could be used as an ocular biomarker alongside current AD diagnostics for evaluating preclinical AD.

Recent studies support the idea of vascular abnormalities and vascular Aβ deposits in retinas of AD patients (Berisha et al., 2007; Liu et al., 2009; Feke et al., 2015; Shi et al., 2020). Aβ plaques were seen in Tg2576 mouse (Alzheimer’s transgenic mice) retinas with increased retinal microvascular deposition of Aβ and neuroinflammation (Liu et al., 2009). Retinal Aβ deposits decreased when Aβ vaccinations were given; however, there was a rise in retinal microvascular Aβ deposition and local neuroinflammation (Liu et al., 2009). This was associated with disruption of retinal organization, therefore, suggesting that retinal imaging could provide a potentially useful non-invasive imaging tool to diagnose AD as well as to observe disease activity/progression (Liu et al., 2009).

Although pericyte loss is a prominent feature of the blood–brain-barrier breakdown in AD, which could be used to monitor cognitive decline, it is not a well-studied area (Shi et al., 2020). One study (Shi et al., 2020) specifically measured and quantified retinal Aβ42 and Aβ40 in retinal blood vessels of AD patients, which was associated with retinal platelet-derived growth factor receptor-β (PDGFRβ). They also found significant Aβ deposits in retinal microvasculature and pericytes in AD, accompanied by substantial pericyte loss (Shi et al., 2020). This study had a good sample size of 56 human donors with AD (post-mortem retinas), which were compared to cognitively normal controls. This study shows possible new targets for AD diagnosis and drug therapy (Shi et al., 2020).

Retinal vascular density is lower in patients with AD and early cognitive impairment (Bulut et al., 2018; Yoon et al., 2019; Zhang et al., 2019). AD patients had a significantly reduced macular vessel and perfusion density compared to controls and individuals with MCI (Yoon et al., 2019). This study’s key strength is that it is the largest prospectively imaged cohort using optical coherence tomography angiography (OCTA). Results can be affected by scan quality; however, a strict quality adherence policy was used in this study. The AD, MCI, and controls were similar in age.

It is important to note that the OCTA technology used in this study is more advanced than traditional fluorescein angiography (FA) in capturing vasculature within the optic nerve head and macula, and it is non-invasive (Zabel et al., 2019b). This is because OCTA provides information on the layers of retinal vasculature separately as opposed to the two-dimensional images offered by FA (De Oliveira et al., 2020). OCTA is being used to analyze the foveal avascular zone (FAZ) as OCTA can detect the FAZ size in more detail than FA; hence OCTA could potentially be used to aid AD ocular diagnosis. The fovea is surrounded by a region completely lacking in retinal capillaries, this region is the FAZ. The FAZ size is related to vision (Shiihara et al., 2018), and in one study (Zabel et al., 2019b), OCTA was used to analyze the FAZ in patients with AD. The study found that when compared to the HC group, participants in the AD group had the biggest mean FAZ area. However, authors of other studies (Van De Kreeke et al., 2019; Salobrar-Garcia et al., 2020), have not found significant differences in FAZ size between HCs and the AD group. Similarly, a meta-analysis of nine studies from a recent review noted that the FAZ was larger in AD groups compared to controls, however, the results were not statistically significant (Jin et al., 2021).

Another study detected a significant decline in vessel density and parafoveal flow in individuals with early cognitive impairment associated with AD, showing it could help the early diagnosis of AD (Zhang et al., 2019). There was a significant correlation between all vascular density parameters and the mini-mental state examination (MMSE; Bulut et al., 2018). The MMSE is a standardized dementia assessment test, however, some people believe it may not be the best measure of AD severity (More et al., 2019). A significantly lower retinal vascular density was detected in AD patients compared to controls. The MMSE score (16.9) for the AD patients was lower in this study compared to the other studies (23.0 and 20.1, respectively); consequently, they may have had more severe AD (den Haan et al., 2019a; Yoon et al., 2019).

Conversely, another study showed no association between MMSE score, CSF biomarkers and retinal vasculature, and there were no significantly detectable differences between AD patients and controls (den Haan et al., 2019a). This study used a small sample size and did not collect all relevant vascular markers. MRI, CSF, and PET imaging were used to define the AD patients; unfortunately, these values were not available for all patients and controls. All the other studies only used a clinical diagnosis, so the groups may not have been defined. Importantly, ophthalmological conditions were controlled for. The AD patients were, on average, 7.7 years younger in this study than the other studies (Zabel et al., 2019a).

Due to the easy access and usage of retinal photography, observing retinal vasculature allows population-wide screening. Other than a narrowing of retinal microvasculature, other measurable aspects of vascular dysfunction for this biomarker include attenuated vessels, reduced branching pattern complexity of vessels and reduced tortuous venules (Frost et al., 2013b).

## Functional Visual Markers

### Visual Acuity

Even though research has found that between AD patients and the control healthy subjects, there is no significant difference in visual acuity (Polo et al., 2017; Colligris et al., 2018; Singh and Shilpa, 2020), there is reduced visual acuity under low luminance in AD patients (Salobrar-García et al., 2019; Singh and Shilpa, 2020). Also, in AD patients, there is a higher risk of cataracts and the capability to identify pictures in reduced spatial frequency is hindered (Singh and Shilpa, 2020).

A large cohort follow up study of over 15,000 older adults without Dementia, showed poorer visual acuity at baseline was associated with higher dementia incidence in 6 years, even after adjusting for demographics, health problems, and lifestyle behaviors, and excluding those who developed dementia within 3 years after baseline. Moderate-to-severe visual impairment could be a potential predictor and possibly a risk factor for dementia (Lee et al., 2020).

### Visual Sensitivity

Alzheimer’s disease patients have significantly decreased contrast sensitivity when compared to the control group, and this difference can be observed during the early stages of AD (Singh and Shilpa, 2020). This is great as it shows potential to be used as an early AD diagnostic tool. In addition, reading speed is reduced in the AD population, and the reading latency is increased (Singh and Shilpa, 2020). Studies have found that visual contrast sensitivity is significantly lower at all spatial frequencies tested in mild and moderate AD groups compared to HCs (Salobrar-García et al., 2019). Donepezil is an anticholinesterase inhibitor that is able to improve contrast sensitivity in AD patients, which is important as in AD patients, the quality of life is negatively impacted by a reduction in visual sensitivity (Singh and Shilpa, 2020). A large proportion of visual acuity and sensitivity depend on foveal thickness; however, a significant reduction in foveal thickness is not observed in AD patients (Polo et al., 2017). The decrease in visual acuity and sensitivity in AD patients could be caused due to the loss of the retinal ganglion cells, resulting in an alteration of the visual pathways (Polo et al., 2017). This suggests that the macular ganglion cells layer could be used as a biomarker for neural damage and AD disease progression (Polo et al., 2017).

### Stereopsis and Depth Perception

The awareness of the distances of objects from the person observing is known as stereopsis or depth perception (Lee et al., 2015). When compared to the control group, AD patients have a reduction in stereopsis and depth perception of three-dimensional structures (Dehghani et al., 2018; Singh and Shilpa, 2020). This is key to note as there is a link between cognitive assessment scores and stereopsis performance (Singh and Shilpa, 2020). To investigate stereopsis depth perception in AD patients is difficult as the patients need to understand the task; therefore, non-depth visual tasks are needed to ensure there is no general comprehension or visual impairment (Bridge, 2016). A study of stereo-acuity tests in AD and healthy subjects found that despite their results not being statistically significant, they fit in with findings of previous and similar literature as they found that stereopsis was impaired in AD patients when compared to HCs (Kim and Lee, 2021). They also found that in assessing stereopsis, three-dimensional TV tests can be more effective than the usual stereo acuity tests (Kim and Lee, 2021). Studies have shown that in AD patients, the weakness in stereopsis results from a decline in the perception of binocular disparity due to impaired functions of the cerebral cortex, such as the visuospatial function (Lee et al., 2015). It is suggested that cognitive function is a factor that also affects stereopsis, suggesting that test tools need to be developed to measure stereopsis effectively and accurately in AD patients, and the appropriate treatments are also needed (Lee et al., 2015).

## Conclusion

With the advent of positron emission tomography (PET) imaging and CSF biomarkers (Aβ and tau), there have been significant advances in the diagnosis of AD and these procedures can identify early neuropathologic changes before the clinical and cognitive decline. However, the clinical diagnosis of MCI or early dementia is challenging due to the insidious onset of disease and gradual cognitive decline (National institute on aging., 2020). In 2011, the National Institutes of Health and the Alzheimer’s Association revised the clinical diagnostic criteria for AD and to showcase a deeper understanding of the disease, they modified research guidelines for the earlier stages of AD (National institute on aging., 2021). However, using biomarkers to assess preclinical AD and increase the certainty of MCI and dementia due to AD are to be used only for research. Before doctors can use these guidelines in clinical practice, more research is needed to make certain biomarkers help predict who will or will not develop Alzheimer’s dementia. Biomarker tests also must be standardized to ensure they can be measured correctly and consistently in all clinical settings (Albert et al., 2011; Clifford et al., 2011; Sperling et al., 2011; McKhann, 2012).

It is well established that there is a global need for a more accurate and earlier diagnosis of AD. Currently, “gold standard” techniques such as PET are expensive and invasive, making population-wide screening difficult. Ocular biomarkers are a novel area for AD diagnosis as the eye has many similarities, both neural and vascular, with the brain. This review focused on three main ocular biomarkers for AD; RNFL is thinning, Aβ deposits and changes in retinal vascular parameters such as narrowing of the vessel and reduced blood flow. Recently, the literature suggests a need for earlier diagnosis and, hence, AD’s treatment due to the evidence that retinal alterations occur early. Using the retina is an easy, non-invasive way to diagnose and manage AD when it is still in the early stages. Some studies have evidenced that only Aβ deposits in the retina appear to be AD specific; however, as there are many ocular biomarkers for AD, more research into each biomarker against AD solely is required. Some retinal vasculature parameters are associated with AD. However, more research is needed to examine the temporal relationship between vascular damage and cognitive decline (Zhang et al., 2019). Many studies have been limited by their study size and cross-sectional design. There is an increasing need to conduct large longitudinal studies that follow pre-symptomatic individuals over time to establish the positive predictive value of retinal findings with AD development.

Many studies observe the effects of AD and dementia; however, AD is only a subtype of dementia; thus, it is crucial not to use the two synonymously. Additional studies that use standardized methods are required to look at how the brain changes parallel with changes in the retina by simultaneously assessing the retina and brain during AD progression (Hadoux et al., 2019). Despite the differences within the literature, it is evident that the retina is affected post-mortem in AD patients and animal models. Some novel imaging techniques include OCT, polarization-sensitive OCT, spectral-domain OCT and cSLO. Even though these techniques are considered novel tests, they need to be refined. When assessing the vascular network, there needs to be an improvement in the histology detail to ensure assessment accuracy. Although ocular biomarkers are heavily researched for their potential non-invasive diagnostic ways, they could also be manipulated to monitor AD treatment progression and whether these biomarkers will reduce if the drug/treatment is effective.

In an era of emerging therapies for AD, there is renewed interest in identifying a reliable biomarker of the disease that can be measured at the point of care. While preliminary evidence suggests that Aβ may accumulate in the lenses and retinas of people with AD, no ocular biomarker of the disease has yet been clinically validated. It is also important to note the lack of specificity of many of the ocular biomarkers such as RNFL thinning and vascular changes, as they can also be seen in other ocular pathologies such as glaucoma. Perhaps a single diagnostic or prognostic biomarker for AD detection may not be achievable for now, but rather using a range of retinal biomarkers may be useful.

## Author Contributions

AM, BM, and HY wrote the manuscript as part of their final year project as part of “Medical Science” degree. HF and MT wrote, reviewed, and revised the manuscript. MT designed, conceived the study, wrote the major revision, made comments, had full access to all data, is the guarantor, and supervised AM, BM, HY. All authors provided important intellectual input and approved the final version of the manuscript.

## Conflict of Interest

The authors declare that the research was conducted in the absence of any commercial or financial relationships that could be construed as a potential conflict of interest.

## Publisher’s Note

All claims expressed in this article are solely those of the authors and do not necessarily represent those of their affiliated organizations, or those of the publisher, the editors and the reviewers. Any product that may be evaluated in this article, or claim that may be made by its manufacturer, is not guaranteed or endorsed by the publisher.

## Abbreviations

Aβ: amyloid-beta
AD: Alzheimer’s disease
APP: amyloid precursor proteins
CNS: central nervous system
cSLO: confocal scanning laser ophthalmoscopy
GCL: ganglion cell layer
IVCCM/CCM: *in vivo* corneal confocal microscopy
MCI: mild cognitive impairment
MMSE: mini-mental state examination
MTA: medial temporal lobe atrophy
NFT: neurofibrillary tangles
OCT: optical coherence tomography
OCTA: optical coherence tomography angiography
PET: Positron emission tomography
pTau: Hyperphosphorylated tau
RGCL: retinal ganglion cell layer
rHSI: retinal hyperspectral imaging
RNFL: retinal nerve fiber layer
RT: retinal thickness
SD-OCT: spectral-domain optical coherence tomography.
